# Hydrogen Sulfide and Substance P Levels in Patients with *Escherichia coli* and *Klebsiella pneumoniae* Bacteraemia

**DOI:** 10.3390/ijms23158639

**Published:** 2022-08-03

**Authors:** Sumeet Manandhar, Amy Scott-Thomas, Michael Harrington, Priyanka Sinha, Anna Pilbrow, Arthur Mark Richards, Vicky Cameron, Madhav Bhatia, Stephen T. Chambers

**Affiliations:** 1 Department of Pathology and Biomedical Science, University of Otago, Christchurch 8140, New Zealand; sumeet.manandhar@postgrad.otago.ac.nz (S.M.); amy.scott-thomas@otago.ac.nz (A.S.-T.); priyanka.sinha@intergen.co.nz (P.S.); steve.chambers@otago.ac.nz (S.T.C.); 2 Microbiology Department, Canterbury Health Laboratories, Christchurch 8140, New Zealand; michael.harrington@cdhb.health.nz; 3 Department of Medicine, University of Otago, Christchurch 8140, New Zealand; anna.pilbrow@otago.ac.nz (A.P.); mark.richards@cdhb.health.nz (A.M.R.); vicky.cameron@otago.ac.nz (V.C.)

**Keywords:** hydrogen sulfide, substance P, sepsis, *Escherichia coli*, *Klebsiella pneumoniae*

## Abstract

Hydrogen sulfide (H_2_S) and substance P (SP) are known from animal models and in vitro studies as proinflammatory mediators. In this study, peripheral blood concentrations of H_2_S and SP were measured in patients with *Escherichia coli* or *Klebsiella pneumoniae* bacteraemia. Fifty patients were recruited from general wards at Christchurch Hospital, during 2020–2021. Samples from age- and sex-matched healthy subjects previously recruited as controls for studies of cardiovascular disease were used as controls. The concentrations of H_2_S were higher than controls on day 0, day 1, and day 2, and SP was higher than controls on all 4 days. The concentrations of H_2_S were highest on day 0, whereas SP concentrations were higher on day 2 than other days. Interleukin-6 and C-reactive protein were significantly higher on day 0 and day 1, respectively. The concentrations of H_2_S and SP did not differ between 15 non-septic (SIRS 0-1) and the 35 septic subjects (SIRS ≥ 2). Substance P concentrations were higher in subjects with abdominal infection than urinary tract infections on day 0 (*p* = 0.0002) and day 1 (*p* = 0.0091). In conclusion, the peak H_2_S concentrations precede the SP peak in patients with Gram-negative bacteraemia, but this response varies with the site of infection.

## 1. Introduction

*Escherichia coli* and *Klebsiella* species are closely related Gram-negative bacilli responsible for human infections and one of the most common causes of infection in hospitalized patients in New Zealand [1] and internationally [2]. These organisms are responsible for intra-abdominal and urinary tract infections and may precipitate sepsis or severe sepsis and have significant morbidity and mortality [3,4]. An increasing proportion of clinical isolates of *E. coli* and *Klebsiella* species are reported to be multi-resistant, including to beta lactam antibiotics, through the acquisition of extended-spectrum beta lactamase and carbapenemase enzymes [5,6]. Consequently, WHO has classified the development of new therapeutic approaches as critical and a priority [7,8]. This adds urgency to the investigation of new approaches that can mitigate the problem of antibiotic resistance and reverse Gram-negative-induced sepsis.

Hydrogen sulfide (H_2_S) and substance P (SP) are well-known inflammatory mediators. H_2_S and SP have been shown to have a proinflammatory role in animal models of experimental sepsis [9,10,11,12,13,14], and there is mounting evidence that H_2_S plays a role in human infections. H_2_S concentrations have been reported to be higher in patients with sepsis and septic shock in ICU than patients with similar physiological disturbance from non-infective insults [15], and in exhaled breath from septic neonates and children compared to controls [16]. Patients with sepsis from *Pseudomonas aeruginosa* lung infections also have elevated H_2_S concentrations in peripheral blood and, in addition, those surviving sepsis from *Pseudomonas* lung infections had higher concentrations of H_2_S than non-survivors. A protective role for elevated H_2_S concentrations was supported by reports of improved survival in a mouse model of *P. aeruginosa*-induced sepsis [17].

Studies of SP in humans with sepsis lack consistency. In ICU patients with sepsis, serum SP concentrations have been reported to be increased during the first week of sepsis [15] and higher concentrations have been reported in survivors than non-survivors [18]. In addition, lower SP levels in the plasma of both septic and septic shock patients compared with control subjects for all time points analysed (onset, 12 h, and 24 h) have been reported [19]. In post-operative patients, those with sepsis had higher SP concentrations than non-septic patients, although higher SP levels were associated with increased mortality in the late stages of sepsis [20].

In a recent study, we highlighted the translational potential of both H_2_S and SP in patients with sepsis via a clinical study where higher circulating levels of H_2_S (12 and 24 h after admission) and SP (48 h after admission) were associated with the inflammatory response but not in patients with related organ failure unrelated to sepsis [15]. These results suggest that alterations in circulating H_2_S and SP concentrations may play an important role in the pathogenesis of sepsis and raises the possibility that the earlier peak in H_2_S concentrations may influence subsequent SP expression. This study also suggested that H_2_S and/or SP could be a potential therapeutic target for clinical sepsis. However, the ICU study included only very unwell patients suffering from diverse infections with a variable clinical course prior to admission to the ICU. In this study, the primary objective was to determine the concentrations of H_2_S and SP in a cohort of patients admitted acutely to hospital with Gram-negative bacteraemia from either *E. coli* or *Klebsiella pneumoniae* and investigate the relationship between these concentrations to the clinical severity of sepsis at admission. The causes of bacteraemia are complex. Most originate from either ascending infection within the urinary tract causing pyelonephritis, or abdominal pathology such as biliary large bowel disease.

## 2. Results

### 2.1. Demographic Characters of Subjects at Presentation

In total, 50 subjects who met the inclusion criteria (see the Methods section) were recruited from 215 patients notified with *E. coli* or *Klebsiella* bacteraemia were approached (23%) for this study (Table 1). Thirty-four subjects had urinary tract infection, including one with ureteric stones. Sixteen subjects had an abdominal source of infection, including cholangitis (10), abdominal perforation (1), abdominal pain cause not identified (2), post biopsy (1), and two patients with neutropenic infection. The demographics and clinical characteristics of those recruited are presented in Table 1. 

On admission, 15 subjects had a systemic inflammatory response syndrome (SIRS) score of 0 or 1, 35 had sepsis with a score of ≥2, and 24 had an early warning score (EWS) of 0–3 and 26 had a score of ≥4.

Subjects were discharged from hospital by the clinical teams as they recovered. The median hospital stay was 4 days (IQR 3–6.3, range 2–26). Four patients (8%) were admitted to intensive care. One subject (2%) died within 30 days of admission. 

### 2.2. Plasma H_2_S, SP, IL-6, and CRP Concentrations and Time Course

**Figure 1:**
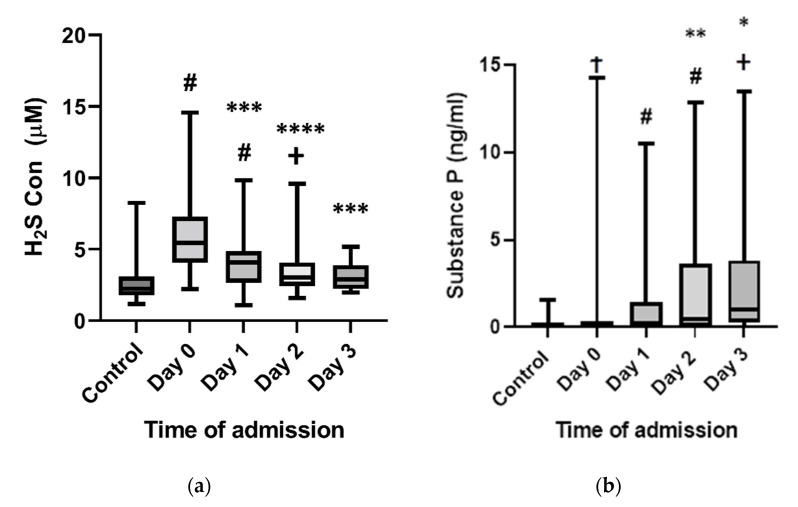
Plasma levels of H_2_S and SP in *E. coli*- and *K. pneumoniae*-infected patients. Plasma samples were collected from patients at different time intervals (days 0, 1, 2, day 3). Controls were healthy age- and sex-matched subjects. Plasma H_2_S (**a**) and SP (**b**) concentrations on days 0, 1, 2, and 3 from patients were compared with each other and non-septic control subjects. Results are expressed as the median with the interquartile range (box plot) with the maximum and minimum value (whiskers plot). Two-tailed paired Mann–Whitney tests were performed. # *p* < 0.0001 vs. control; + *p* < 0.01 vs. control; † *p* < 0.05 vs. control; **** *p* < 0.0001 vs. day 0; *** *p* < 0.001 vs. day 0; ** *p* < 0.01 vs. day 0; * *p* < 0.05 vs. day 0.

Plasma H_2_S and SP concentrations in bacteraemic subjects are shown in Figure 1a,b. The median concentrations of H_2_S in subjects were higher than controls (*n* = 50) on days 0, 1, and 2 (*p* < 0.0001, *p* < 0.0001, *p* = 0.0075, respectively) but not on day 3 (*p* = 0.09). Day 0 concentrations of H_2_S (*n* = 44) were higher than days 1 (*n* = 40, *p* = 0.0011), 2 (*n* = 27, *p* < 0.0001), and 3 (*n* = 10, *p* = 0.003). Concentrations on day 1 were not significantly higher than on days 2 and 3 (*p* = 0.051 and 0.09), respectively. 

The median concentrations of SP in subjects were higher than controls (*n* = 50) on days 0, 1, 2, and 3 (*p* < 0.007, *p* < 0.0001, *p* = 0.0001, *p* < 0.001 respectively).

**Figure 2:**
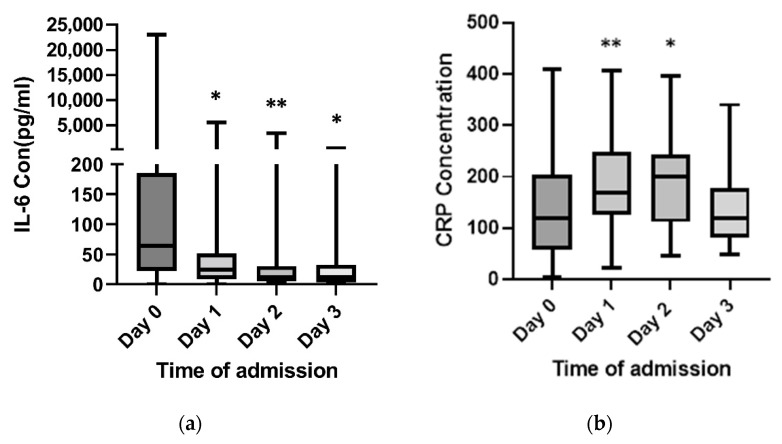
Plasma levels of IL-6 and CRP in *E. coli*- and *K. pneumoniae*-infected patients. Plasma samples were collected on days 0, 1, 2, and 3. Plasma IL-6 (**a**) and CRP (**b**) levels on days 1, 2, and 3 in patients are compared with the day 0 results. Results are expressed as the median with the interquartile range (box plot) with the maximum and minimum value (whiskers plot). Two-tailed paired Mann–Whitney tests were performed. ** *p* < 0.01 vs. day 0; * *p* < 0.05 vs. day 0.

The median concentrations of SP on day 0 (*n* = 44, IQR 0.0–0.42) did not differ from day 1 (*n* = 41, *p* = 0.55) but were higher on days 2 (*n* = 25, *p* < 0.0001) and 3 (*n* = 9, *p* = 0.0002). Interleukin-6 concentrations were highest on day 0 (*n* = 38) compared to days 1 (*n* = 40, *p* = 0.0401) and 2 (*n* = 29, *p* = 0.0036). C-reactive protein (CRP) was higher on days 1 (*n* = 40, *p* = 0.007) and 2 (*n* = 40, *p* = 0.0149) compared to day 0 (*n* = 46) (Figure 2a,b).

The day 0 H_2_S concentration correlated with the concentration of SP on day 2 (*p* = 0.022) while the other correlations did not reach significance. 

There was insufficient data (<8 samples) on days 4 and 5 for analysis, as most patients were recovered and discharged after day 3. 

### 2.3. Comparison of Plasma H_2_S, SP, IL-6, and CRP Concentrations in Urinary and Abdominal Infection

Because of the lack of a relationship between the severity scores and the levels of H_2_S and SP, the data was analysed by site of infection. There was no significant difference in the age, gender, systemic inflammatory response syndrome (SIRS), early warning score (EWS), CRP, or H_2_S concentrations between the urinary and abdominal infection groups (Table 2). However, SP concentrations were significantly lower in the urinary infection groups compared to abdominal infection on days 0 (*p* = 0.0002) and 1 (*p* = 0.0091).

In the urinary tract infection group, there was a significant correlation between the SIRS score and H_2_S concentration on days 0 (*p* = 0.034) and 1 (*p* = 0.037), and between the EWS score and H_2_S concentration on days 0 (*p* = 0.036) and 1 (*p* < 0.0005) (Supplementary Table S1b). There was a significant correlation between the SP concentration and SIRS (*p* = 0.02) and EWS (*p* = 0.033) on day 0 only.

## 3. Discussion

In this study of bacteraemia subjects with the Gram-negative organisms, *E. coli* and *K. pneumonia*, the organisms responsible for most cases of Gram-negative sepsis, we found that the clinical severity on admission (SIRS and EWS) was not related to the measured blood concentrations of either H_2_S or SP.

H_2_S concentrations were most elevated at the time of admission and decreased from day 1 to day 3 after medical intervention. The median SP concentrations followed a different time course and increased progressively over the observations period, although they were higher on days 0 and 1 compared with controls. These results are consistent with our previous ICU study, where H_2_S and SP levels were elevated in sepsis, but are independent of the clinical parameters used to measure severity, such as APACHE II and III, SAPS II, and SOFA [15]. They also followed a similar time course to the changes observed in our ICU study [15,16]. The results add to previous knowledge by showing that H_2_S concentrations may be elevated in bacteraemic but not septic subjects and suggest that this response is triggered as part of an appropriate initial response to infection by the host.

The high SP concentrations on days 2 and 3 may in part reflect the severity of the infection in those remaining in hospital as those who had recovered, and presumably were less severely affected, had been discharged from hospital promptly and were unavailable for sampling. These results are consistent with other studies that have found SP concentrations were elevated during the late stages of lethal sepsis [15,20]. During the first week of admission, surviving septic patients had higher serum SP levels than non-survivors during the first week, suggesting the role of SP as a biomarker of sepsis mortality [18,21].

The comparison of the results between abdominal and urinary tract infections yielded a new perspective. Firstly, there was strong evidence that SP concentrations were higher on both day 0 and day 1 in those with abdominal infection than those with urinary tract infection, although both had similar SIRS and EWS scores. This suggests that the contribution of the SP pathway to the inflammatory response may be influenced by the site of infection, but factors such as the length of time the subjects were unwell before being admitted to hospital or the polymicrobial infections commonly found in abdominal but not urinary tract infections may confound this finding. Secondly, there was evidence that day 0 H_2_S concentrations were correlated with the SIRS and EWS in urinary tract infection but not in abdominal infections. Interestingly, both the EWS and SIRS scores were strongly negatively correlated with day 1 H_2_S concentrations in urinary tract infections, suggesting that H_2_S concentrations may fall quickly in subjects with the most marked early clinical manifestation of infection. Finally, the results are consistent with the observation that elevated SP concentrations are more reflective of ongoing established infection rather than the first phase of an acute response. On day 0, SP concentrations correlated negatively with both the SIRS and EWS in both urinary and abdominal infections but not at other time points. Additionally, SP on day 1 also correlated with the subsequent length of hospital stay in abdominal infections.

The sequence of the early increase in the H_2_S concentration followed by a rise in SP in this and previous clinical studies raises the questions as to whether H_2_S may play a role in triggering the SP response. Previous studies have established H_2_S and SP as proinflammatory mediators in various mouse models [11,21,22,23,24,25,26]. Following cecal ligation and puncture (CLP)-induced sepsis, there was an increase in tissue H_2_S-synthesising activity, plasma H_2_S levels, and tissue and plasma SP levels. H_2_S donor, Na_2_S and NaHS, administration has been demonstrated to elevate local and systemic inflammation, whereas DL-propargylglycine (an inhibitor of cystathionine-γ-lyase H_2_S-synthesizing enzyme) and neurokinin-1 receptor (substance P receptor) antagonist treatment protected against the sepsis-induced systemic inflammatory response and multiple organ dysfunction in mice [11,12,24]. Furthermore, gene knockout of cystathionine-γ-lyase and preprotackykinin (PPTA gene that codes for SP) in mice protected against CLP sepsis that induced local and systemic inflammation [9,10,13,26]. H_2_S upregulated SP synthesis and its circulating blood concentration in a mouse model [23,24,27]. However, genetic deletion of PPTA has no effect on the H_2_S level following CLP-induced sepsis, demonstrating that SP is located downstream of H_2_S in the inflammatory pathway. In addition, SP levels in CLP-induced sepsis were not restored to the level of sham-operated mice, even after the inhibition of H_2_S formation, suggesting that the pathogenesis of sepsis is a complicated system, where the synthesis of SP is influenced by other mediators in addition to H_2_S [28]. In addition, H_2_S and SP have shown direct effects on the expression level of chemokines and cytokines independently of each other in vitro [29,30,31,32]. These studies show that although H_2_S acts via SP in inflammation, these two inflammatory mediators have pathways that may be independent of each other. Our recent clinical study showed that the H_2_S concentration was elevated at both the 12- and 24-h time points while SP levels were elevated from 48 to 96 h [15]. Similarly, in this study, we showed that H_2_S levels increase early (day 0) in the inflammatory response while the SP level increases later (day 2 and day 3), supporting the evidence that H_2_S may be responsible for upregulating SP during sepsis. However, the discrepancy in the SP level, with H_2_S showing alteration of its level relative to the site of infection, raises the question of whether SP is influenced by various pathways other than the H_2_S pathway as shown by our pervious mouse model experiments.

C-reactive protein levels have been of great clinical interest for early diagnosis of infection and monitoring its treatment response as they are easily measured and related to the severity of the systemic inflammatory response [33,34,35,36,37]. The results of the present study show a higher plasma concentration of CRP, peaking on day 1, but there was no significant correlation between the H_2_S and SP concentrations in either the urinary tract infection or abdominal infection group, although this may become apparent in a larger cohort. IL-6 serves as a major mediator during the early phase of the acute response to inflammation in sepsis, and its clinical importance has been assessed in several studies on patients with various septic conditions [38,39,40,41]. The early rise in the IL-6 concentrations was similar to the plasma H_2_S levels. Overall, these results suggest IL-6 and H_2_S may serve as early markers of inflammation during *E. coli*- and *K. pneumoniae*-induced sepsis.

Limitations: Although this study conclusively shows that H_2_S and SP have an important role in patients with Gram-negative bacteraemia, the sample size is an obvious limitation of this study. Further investigation with a larger patient population with well-defined clinical and microbiological infections is required to understand the significance of H_2_S and SP in human sepsis. The methylene blue method (assay), which is used to quantify H_2_S levels, also quantifies other H_2_S-related compounds. This assay was used as it is convenient for quantifying many clinical samples and has been used in animal studies with success. The use of methods with higher specificity for H_2_S would be advantageous.

In conclusion, this translational study demonstrated that higher concentrations of H_2_S correlated with the very early and SP with the later phase of the inflammatory response in *E. coli* and *K. pneumoniae* bacteraemia and suggests there may be variation in the pathway of activation of the inflammatory response between urinary tract and abdominal infection. This has important implications for future clinical research of H_2_S and SP in the ICU, where infection at multiple sites, including the lung, skin and soft tissue, and bone and joint infection, may be included with abdominal and urinary tract infection. Caution should also be used when interpreting the results of animal studies of single-site infections to avoid overgeneralising the systemic implications of this study. Further studies are needed to determine the potential role of the H_2_S and SP pathways to assess patient severity and novel therapeutic approaches for these patients.

## 4. Materials and Methods

### 4.1. Study Design and Selection of Subjects

This study was an observational prospective prognostic cohort study of patients admitted to Christchurch Hospital, Christchurch, New Zealand with *E. coli* or *K. pneumoniae* bacteraemia. Christchurch Hospital provides secondary and tertiary care for the Canterbury area of New Zealand, serving a population of about 550,000 people. The protocol, participant informed consent form, participant information sheet, family/whānau information sheet, family/whānau consent form, and questionnaires were approved by a Human Health and Disability Ethics Committee (Ethics ref: 20/STH/5). Written informed consent was provided by all patients who participated in this study. Patients who were confused, admitted during the weekend, or discharged within 24 h were not able to be approached to take part in this study.

#### Procedures

Patients admitted to hospital with clinical suspicion of sepsis such as signs and symptoms of acute infection and an elevated body temperature had blood cultures drawn as part of their clinical work up. Temperature, respiratory rate, heart rate, blood temperature, and oxygen saturation were recorded every 6–8 h onto an electronic database by clinical staff.

Blood cultures and clinical specimens for routine haematological, biochemical, and microbiological studies were processed at the clinical laboratory (Canterbury Health Laboratories). Blood was cultured using an automated culture system (Bactec SX). Antimicrobial susceptibility was measured with a BD Phoenix^TM^ instrument to determine the minimum inhibitory concentrations. Antimicrobial therapy was administered by the clinical team responsible for the patient according to the hospital guidelines. Investigators were notified of positive blood cultures with a Gram-negative organism online as soon as they became positive and were approached for suitability consent for enrolment.

The inclusion criteria were:

Any patient with *E. coli* or *K. pneumoniae* isolated from the blood or sterile site (e.g., joints) with or without sepsis’

Aged ≥ 18 years;

Able to provide informed consent;

Investigators notified within 24 h of admission.

The exclusion criteria were:

All patients with sepsis caused by other organisms. If it was a polymicrobial infection, cases were included as long one organism was identified as *E. coli* or *K. pneumoniae*;

COVID-related restrictions that denied access to the hospital for research studies;

Patient unable to provide informed consent.

### 4.2. Definitions

Bacteraemia was defined as isolation of *E. coli* or *K. pneumonia* from one or more sets of blood culture. Two sets of blood cultures were taken routinely on admission, although further blood cultures were taken at the discretion of the clinical team responsible for their care.

Sepsis was defined as a systemic inflammatory response syndrome (SIRS) score of >2 [42] or an early warning score (EWS) of >4 based on [43,44], with the highest recorded in the first 24 h of admission to hospital [45,46]. The qSOFA score could not be measured as patients with confusion were excluded.

### 4.3. Blood Collection and Plasma Preparation

#### 4.3.1. Day 0 Samples

Blood samples for biochemical and haematological analyses collected on admission for patient evaluation were separated and stored in a refrigerated auto-analysing suite. Residual ethylene-diamine-tetra-acetic acid (EDTA) blood samples of newly enrolled subjects were retrieved daily. A pilot study showed H_2_S and SP are stable in this system and suitable for retrieval and analysis within 24 h.

#### 4.3.2. Follow-Up Samples

Blood was taken daily while the subjects were in hospital for up to 5 days. Blood samples were drawn through venepuncture and collected into 6-mL EDTA vacutainer tubes and placed on ice for transport to the laboratory, and centrifuged at 1000× *g* for 10 min at 4 °C. Clear plasma was collected and aliquoted into multiple tubes. Assays for C-reactive protein (CRP) were performed as per the established protocols at Canterbury Health Laboratory, Christchurch, New Zealand. Remaining plasma was stored as aliquots at –80 degrees C. An individual aliquot was used for each analysis, which was carried out in batches.

#### 4.3.3. Control Samples

These were selected from an archive of 3358 biobanked samples from patients recruited as controls for studies of cardiovascular disease (CVD), who also consented for their samples to be used in other studies (Trial Registry ACTRN1260500448640) [47]. This study was approved by the Upper South A Ethics Committee (Reference No. CTY/01/05/062), and each participant provided written informed consent. The inclusion criteria were that they were clinically well and had no history or hospital admissions for CVD, no self-reported history of diabetes (type 1 or type 2), hypertension, high cholesterol, joint disease, gut disease, skin disease, thyroid disease, asthma, or cancer. One to one matching was performed using the MatchIt package (https://github.com/kosukeimai/MatchIt, accessed on 28 February 2022) [48] in R [49], which identified 50 subjects matching for age (median 72.5 for bacteraemia patients and 71.8 for controls (*p* = 0.927)), gender (*p* = 0.834), and ethnicity.

### 4.4. Measurement of Plasma H_2_S Concentration

Hydrogen sulfide was measured using a spectrophotometric assay (methylene blue method) as described by Gaddam et al. [15]. In brief, plasma samples were mixed with equal volumes of phosphate buffer and a mixture of pyridoxal phosphate L-cysteine, zinc acetate, N,N-dimethyl-p-phenylenediamine sulphate, and FeCl_3_HCl. The mixture was incubated in the dark for 20 min at room temperature after which trichloroacetic acid was added to denature the protein and stop the reaction. Samples were centrifuged at 7700× *g* for 5 min at 4 °C and 150 µL supernatant transferred into a 96-well microplate. Absorbance was measured at 670 nm (Multiskan Go, Thermofisher). H_2_S was calculated using a calibration curve of sodium sulfide (0.4–25 µM) and H_2_S levels were expressed as µM.

### 4.5. Measurement of Plasma Substance P Concentration

Human plasma SP concentrations were measured using ELISA (Bachem; Peninsula Laboratories, Bubendorf, Switzerland) according to the manufacturer’s protocol. Absorbance was measured at 450 nm by a spectrophotometer (Multiskan Go, Thermofisher, Vantaa, Finland). SP levels were expressed as ng per 100 µL plasma.

### 4.6. Measurement of Plasma Interleukin-6 Concentration

Plasma IL-6 levels were measured using sandwich ELISA Duoset kits (R&D systems, Minneapolis, MN, USA) according to the manufacturer’s protocol. Assay absorbance was measured at 450 nm on a spectrophotometer (Multiskan Go, Thermofisher) with 540 nm correction. The IL-6 concentration was expressed as pg/mL in plasma.

### 4.7. Statistical Analysis

Continuous variables were summarized with descriptive analyses that included medians, ranges, and interquartile ranges where appropriate. Categorical variables are given as frequencies and percentages. Exploratory hypothesis testing was carried out using the Mann–Whitney U test. Statistical analyses were performed by GraphPad software (version 9, Prism, San Diego, CA, USA). Comparison between groups used the two-tailed Mann–Whitney U test and Spearman coefficient for correlations. A *p*-value of less than 0.05 was considered as statistically significant.

## Figures and Tables

**Table 1:** Demographic characteristics of subjects admitted to Christchurch Hospital during the 2020–2021 study period. Data are expressed as the median with the interquartile range unless otherwise stated.

Patient Characteristics	Patients	Controls
	*n* (%) *	*n* (%)
Age—years	72.5 (29–90)	71.8 (37–86)
Female	33 (66%)	32 (64%)
European	44 (88%)	46 (92%)
Māori	2 (4%)	2 (4%)
Asian	2 (4%)	2 (4%)
Other	2 (4%)	0 (0%)
Charlson age	3 (0–10)	-
Urinary tract infection	34 (68%)	0 (0%)
Abdominal infection	16 (32%)	0 (0%)
Neutrophils × 10^9^/L	13.85 (0–28.6)	-
CRP mg/L	118.5 (5–409)	-
Creatinine mg/L	98 (56–734)	97 (75–136)
eGFR mL/s	52.5 (6–99)	65 (46.5–93)
EWS ^	4 (0–14)	-
SIRS ^^	2 (0–4)	-
Co morbidities **		
Neutropenia ***	2 (4%)	-
Cardiovascular disease	13 (26%)	0 (0%)
Cerebrovascular disease	6 (12%)	0 (0%)
Congestive heart failure	3 (6%)	0 (0%)
Chronic obstructive pulmonary disease	6 (12%)	-
Connective tissue disease	6 (12%)	-
Peptic ulcer disease	6 (12%)	-
Solid tumour	12 (24%)	0 (0%)
Liver disease	2 (4%)	-
End-stage renal failure /kidney transplant	4 (8%)	-
Lymphoma	2 (4%)	0 (0%)
Acute myeloid leukaemia	1 (2%)	0 (0%)

^ Early warning score; ^^ Systemic inflammatory response syndrome; * Unless otherwise stated; ** Patients may have multiple co-morbidities; *** AML 1, Mantel cell lymphoma 1.

**Table 2:** Comparison of plasma H_2_S, SP, IL-6, and CRP concentrations between urinary tract and abdominal infections. The SIRS and EWS scores and white blood cell count were obtained from observations and samples taken during the first 12 h after admission. Data are expressed as the median with the interquartile range unless otherwise stated.

	Day	Abdomen	Urinary Tract	*p* Value
Number		16	34	
Age		73 (62–82)	74 (63–82)	>0.99
Gender		6 (37.5%)	11 (32.4%)	
SIRS		2 (1.3–3)	2 (1–4)	0.8919
EWS		3.5 (1.3–5.8)	4 (2–5.3)	0.7664
White blood cell count × 10^9^/L		11.0 (5.6–15)	14 (10–20)	0.0492
H_2_S	Day 0	6.1 (5.2–8.1)	4.9 (3.3–7.1)	0.0567
	Day 1	4.2 (3.4–4.6)	3.3 (2.5–5.3)	0.2764
	Day 2	3.5 (2.7–4.4)	2.9 (2.2–3.8)	0.2120
	Day 3	3.3 (2.5–4.2)	2.5 (2–4.1)	0.4206
SP	Day 0	0.47 (0.09–2)	0 (0–0.92)	0.0002
	Day 1	0.7 (0.16–3.3)	0.026 (0–0.63)	0.0091
	Day 2	0.7 (0.42–4.1)	0.13 (0.003–4.8)	0.2095
	Day 3	2.2 (0.22–11)	1.1 (0.46–3.2)	0.9048
IL-6	Day 0	49.18 (5.70–290.1	59.23 (22.53–114.1)	0.8911
	Day 1	14.35 (6.36–193.2)	31.99 (11.71–53.66)	0.3395
	Day 2	26.77 (9.18–170.6)	11.41 (4.18–27.21)	0.1816
	Day 3	2.86 (1.24–4.48)	16.76 (5.24–29.77)	0.1905
CRP	Day 0	160 (22–160.5)	148 (71–219)	0.1426
	Day 1	147.5 (123.5–232	187.5 (151.5–274)	0.0871
	Day 2	216.5 (183.5–287)	273.5 (215–397)	0.2190
	Day 3	183.5 (100.5–216.5)	215 (125.5–273.5)	0.6700

## Data Availability

The data presented in this study are available on request from Professor Stephen T Chambers. The data are not publicly available and stored on a secure system at the University of Otago, Christchurch.

## Supplementary Materials

The following supporting information can be downloaded at: https://www.mdpi.com/article/10.3390/ijms23158639/s1.
