# Supplementary material for: Hydrogen Sulfide and Substance P Levels in Patients with Escherichia coli and Klebsiella pneumoniae Bacteraemia
Source: Int J Mol Sci. 2022 Aug 3;23(15):8639. doi: 10.3390/ijms23158639 (PMC9368963; doi:10.3390/ijms23158639)
Supplement: Supplementary file 1 [file ijms-23-08639-s001.zip › ijms-1813735-supplementary.pptx]

## Slide 1
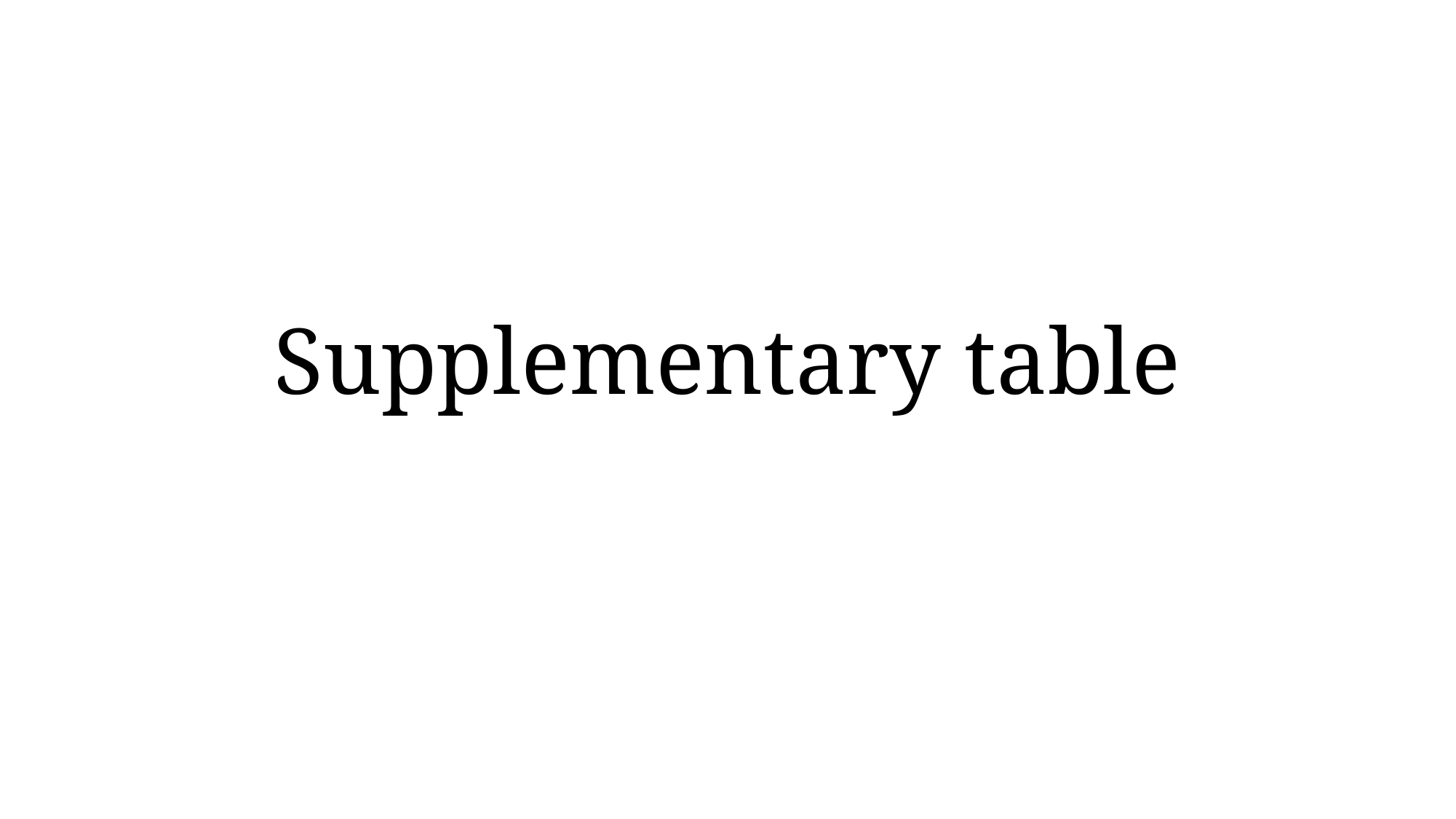

# Supplementary table

## Slide 2
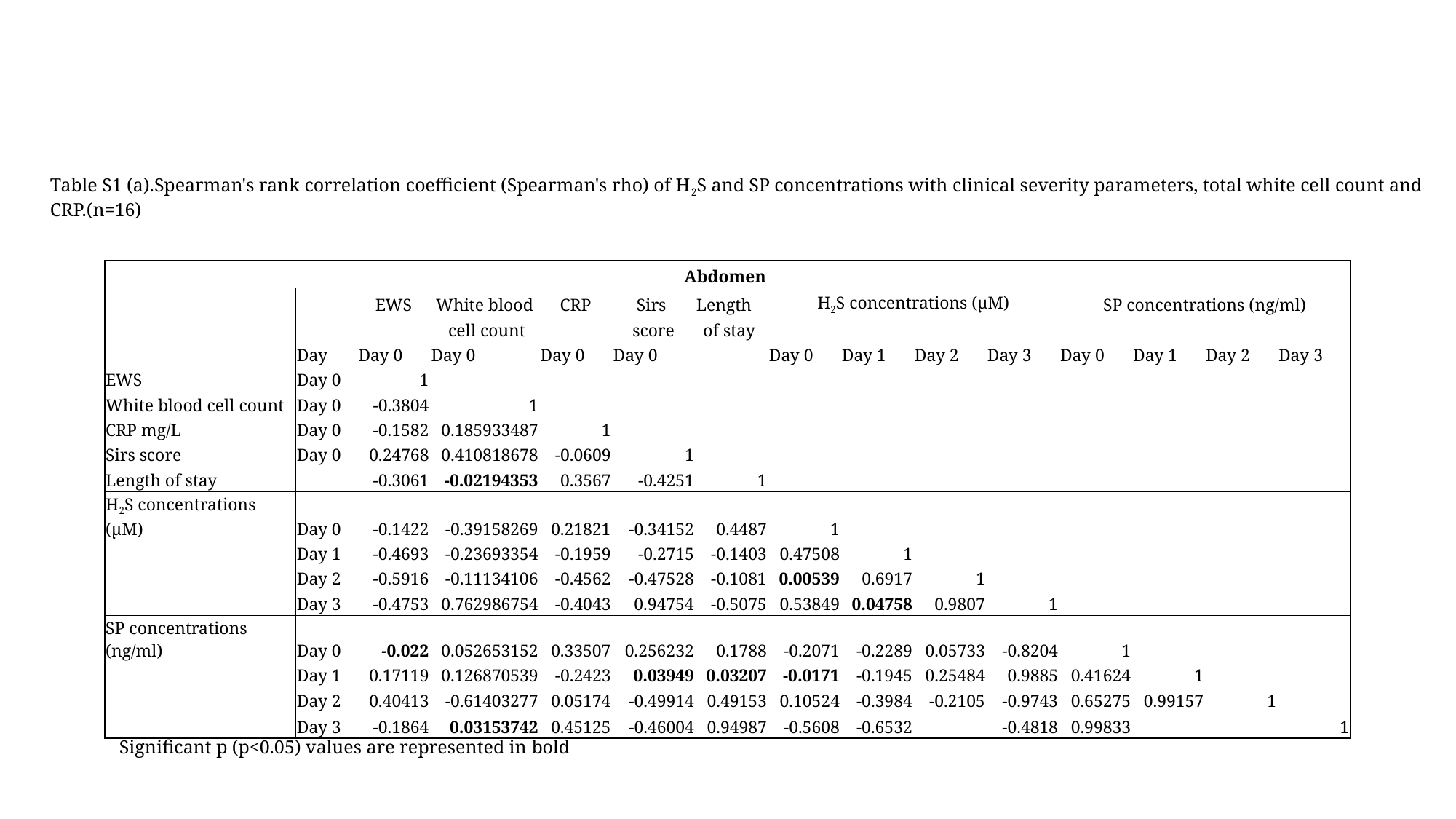

Table S1 (a).Spearman's rank correlation coefficient (Spearman's rho) of H2S and SP concentrations with clinical severity parameters, total white cell count and
CRP.(n=16)
| Abdomen | | | | | | | | | | | | | | |
| --- | --- | --- | --- | --- | --- | --- | --- | --- | --- | --- | --- | --- | --- | --- |
| | | EWS | White blood | CRP | Sirs | Length | H2S concentrations (µM) | | | | SP concentrations (ng/ml) | | | |
| | | | cell count | | score | of stay | | | | | | | | |
| | Day | Day 0 | Day 0 | Day 0 | Day 0 | | Day 0 | Day 1 | Day 2 | Day 3 | Day 0 | Day 1 | Day 2 | Day 3 |
| EWS | Day 0 | 1 | | | | | | | | | | | | |
| White blood cell count | Day 0 | -0.3804 | 1 | | | | | | | | | | | |
| CRP mg/L | Day 0 | -0.1582 | 0.185933487 | 1 | | | | | | | | | | |
| Sirs score | Day 0 | 0.24768 | 0.410818678 | -0.0609 | 1 | | | | | | | | | |
| Length of stay | | -0.3061 | -0.02194353 | 0.3567 | -0.4251 | 1 | | | | | | | | |
| H2S concentrations (µM) | Day 0 | -0.1422 | -0.39158269 | 0.21821 | -0.34152 | 0.4487 | 1 | | | | | | | |
| | Day 1 | -0.4693 | -0.23693354 | -0.1959 | -0.2715 | -0.1403 | 0.47508 | 1 | | | | | | |
| | Day 2 | -0.5916 | -0.11134106 | -0.4562 | -0.47528 | -0.1081 | 0.00539 | 0.6917 | 1 | | | | | |
| | Day 3 | -0.4753 | 0.762986754 | -0.4043 | 0.94754 | -0.5075 | 0.53849 | 0.04758 | 0.9807 | 1 | | | | |
| SP concentrations (ng/ml) | Day 0 | -0.022 | 0.052653152 | 0.33507 | 0.256232 | 0.1788 | -0.2071 | -0.2289 | 0.05733 | -0.8204 | 1 | | | |
| | Day 1 | 0.17119 | 0.126870539 | -0.2423 | 0.03949 | 0.03207 | -0.0171 | -0.1945 | 0.25484 | 0.9885 | 0.41624 | 1 | | |
| | Day 2 | 0.40413 | -0.61403277 | 0.05174 | -0.49914 | 0.49153 | 0.10524 | -0.3984 | -0.2105 | -0.9743 | 0.65275 | 0.99157 | 1 | |
| | Day 3 | -0.1864 | 0.03153742 | 0.45125 | -0.46004 | 0.94987 | -0.5608 | -0.6532 | | -0.4818 | 0.99833 | | | 1 |
Significant p (p<0.05) values are represented in bold

## Slide 3
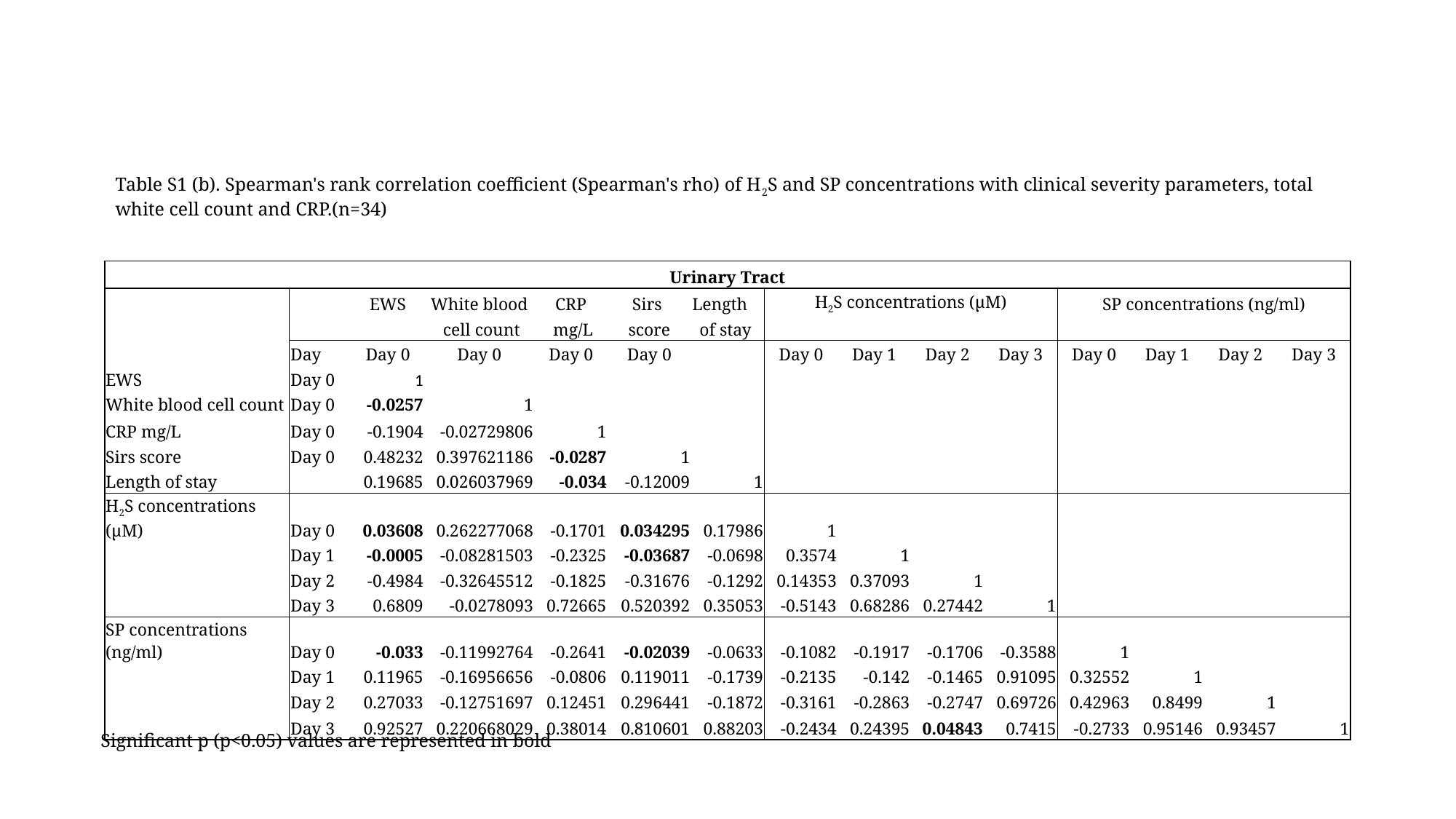

Table S1 (b). Spearman's rank correlation coefficient (Spearman's rho) of H2S and SP concentrations with clinical severity parameters, total white cell count and CRP.(n=34)
| Urinary Tract | | | | | | | | | | | | | | |
| --- | --- | --- | --- | --- | --- | --- | --- | --- | --- | --- | --- | --- | --- | --- |
| | | EWS | White blood | CRP | Sirs | Length | H2S concentrations (µM) | | | | SP concentrations (ng/ml) | | | |
| | | | cell count | mg/L | score | of stay | | | | | | | | |
| | Day | Day 0 | Day 0 | Day 0 | Day 0 | | Day 0 | Day 1 | Day 2 | Day 3 | Day 0 | Day 1 | Day 2 | Day 3 |
| EWS | Day 0 | 1 | | | | | | | | | | | | |
| White blood cell count | Day 0 | -0.0257 | 1 | | | | | | | | | | | |
| CRP mg/L | Day 0 | -0.1904 | -0.02729806 | 1 | | | | | | | | | | |
| Sirs score | Day 0 | 0.48232 | 0.397621186 | -0.0287 | 1 | | | | | | | | | |
| Length of stay | | 0.19685 | 0.026037969 | -0.034 | -0.12009 | 1 | | | | | | | | |
| H2S concentrations (µM) | Day 0 | 0.03608 | 0.262277068 | -0.1701 | 0.034295 | 0.17986 | 1 | | | | | | | |
| | Day 1 | -0.0005 | -0.08281503 | -0.2325 | -0.03687 | -0.0698 | 0.3574 | 1 | | | | | | |
| | Day 2 | -0.4984 | -0.32645512 | -0.1825 | -0.31676 | -0.1292 | 0.14353 | 0.37093 | 1 | | | | | |
| | Day 3 | 0.6809 | -0.0278093 | 0.72665 | 0.520392 | 0.35053 | -0.5143 | 0.68286 | 0.27442 | 1 | | | | |
| SP concentrations (ng/ml) | Day 0 | -0.033 | -0.11992764 | -0.2641 | -0.02039 | -0.0633 | -0.1082 | -0.1917 | -0.1706 | -0.3588 | 1 | | | |
| | Day 1 | 0.11965 | -0.16956656 | -0.0806 | 0.119011 | -0.1739 | -0.2135 | -0.142 | -0.1465 | 0.91095 | 0.32552 | 1 | | |
| | Day 2 | 0.27033 | -0.12751697 | 0.12451 | 0.296441 | -0.1872 | -0.3161 | -0.2863 | -0.2747 | 0.69726 | 0.42963 | 0.8499 | 1 | |
| | Day 3 | 0.92527 | 0.220668029 | 0.38014 | 0.810601 | 0.88203 | -0.2434 | 0.24395 | 0.04843 | 0.7415 | -0.2733 | 0.95146 | 0.93457 | 1 |
Significant p (p<0.05) values are represented in bold
